# Editorial: Current Challenges and Future Developments in Robot Grasping

**DOI:** 10.3389/frobt.2022.973208

**Published:** 2022-07-11

**Authors:** Antonio Morales, Beatriz León, Eris Chinellato, Raúl Suárez

**Affiliations:** ^1^ Department of Engineering and Computer Science, Universitat Jaume I, Castelló de La Plana, Spain; ^2^ Shadow Robot Company, London, United Kingdom; ^3^ Department of Design Engineering and Mathematics, Middlesex University, London, United Kingdom; ^4^ Institute of Industrial and Control Engineering, Universitat Politècnica de Catalunya, Barcelona, Spain

**Keywords:** robotics, grasping, manipulation, robot grasp planning, robot learning

Robot grasping describes a robot’s ability to grasp and manipulate objects which is one of the most desired capabilities for a fully functional robot system in many applications. However, grasping is one of the hardest problems in robotics since it has to deal with complex perception issues, planning and executing difficult and careful interactions, and requires advanced devices and reasoning.

As a consequence, it is a field which has attracted a great deal of interest and research over the last decades, which has resulted in many advances and an active area of research and development. In the last few years, research related to robot grasping has focused on two main aspects. The first is the application of deep learning techniques to robot grasping in order to apprehend and overcome most of the difficult cognitive problems. These applications have not only addressed the perception, but also the planning and control aspects of grasping. The second aspect is the design of novel grippers or robot hands applying principles of soft mechanisms, with the purpose of providing more adaptable and versatile devices. Nevertheless, other more traditional research lines are still present and should not be neglected in a complete view of robot grasping. These include perception, mostly vision but also haptics; planning, which involves grasp synthesis, analysis and metrics; control and specific applications.

The papers collected in this Research Topic provide a good example of the broad spectrum of areas of research that still present a lot of challenges. The first paper, with title “*DGCM-Net: Dense Geometrical Correspondence Matching Network for Incremental Experience-Based Robotic Grasping*,” by Patten et al. presents a method for grasping novel objects by learning from previous successful experiences. The successful grasps are stored in a database, encoding objects with similar geometries close to each other in a feature space. Then, looking for a grasp for a new object is based on a nearest neighbour search in this feature space and an adaptation of the existing grasps to the new object. Specific techniques are developed as part of the contribution to deal with this problem. The approach also allows an improvement in the grasp success rate when experience is accumulated. Real experiments are reported to validate the proposed approach.

The following paper “*Leveraging Human Perception in Robot Grasping and Manipulation Through Crowdsourcing and Gamification*” by Gorjup et al. presents a framework that combines crowdsourcing and gamification to leverage human intelligence, enhancing the object recognition and attribute estimation processes of robot grasping. The framework is aimed to facilitate the assignment of semantic attributes of visual instances of objects. This is done by a crowdsourcing framework where different human subjects collaborate in the recognition of such attributes. The relevant contribution of the paper is the introduction of a gamified interface to ease and motivate the task of the human collaborators. The paper presents two cases that demonstrate the usefulness of the approach: the control of an exoskeleton while manipulating objects and the object recognition and grasping for a dual-arm service robot.

The third paper, with title “*Aiding Grasp Synthesis for Novel Objects Using Heuristic-Based and Data-Driven Active Vision Methods,*” by Natarajan et al., presents an approach to optimise a sequence of viewpoints of an object to efficiently collect the necessary data for the grasp synthesis using a two-finger parallel gripper. The paper describes two optimization methods, based on heuristics and considering a depth camera mounted on the robot arm. The work includes an extensive set of simulated and real experiments to validate relevant statistical results and comparisons with baseline methods.

The next work “*Grasp Stability Prediction for a Dexterous Robotic Hand Combining Depth Vision and Haptic Bayesian Exploration*” also addresses the issue of stability prediction for grasp selection, but from a different point of view. Siddiqui et al. propose a framework for determining stable and safe grasping actions on unknown or un-modelled objects, based on a multimodal visual and tactile exploration. A system composed by camera, articulated arm and dexterous hand with tactile feedback is used in the final robot experiments, building upon simulated tests for initial safety and performance assessment. Visual analysis through an RGB-D sensor is employed to establish initial contact points based on an estimated quality metric on the visible object surface. Tactile exploration is performed starting from the moment the hand touches the object and different exploration methods (standard and unscented Bayesian Optimisation and uniform grid search) are employed to find a stable contact area for the fingers in order to achieve a reliable grip. Results show that probabilistic methods, particularly the unscented Bayesian Optimisation, can provide confident stability predictions after only a few nexploratory observations.

Finally, Meattini et al. on their work “*Simulative Evaluation Of A Joint-Cartesian Hybrid Motion Mapping For Robot Hands Based On Spatial In-Hand Information,*” proposed a way of combining two approaches to improve the control of the robot hands when using teleoperation exploiting the spatial information available in-hand related to the thumb-fingers relative position, for combining joint and Cartesian mappings. This allows to improve the performance of a large range of grasps both volar (where the preservation of finger shapes is more important) and precision grips (where the preservation of fingertip positions is more important). The evaluation was done using simulation of four well-known robot hands showing that both approaches were capable of successfully performing the mapping of finger shapes and fingertip positions, on both anthropomorphic and non-anthropomorphic robot hands. It will be interesting to see in future works the performance of these experiments with real robot hands to investigate the specific telemanipulation improvements using the proposed mapping.

This Research Topic of papers provides a useful insight on recent, diverse, approaches to the realm of robot grasping and its most recent advances. It also outlines important scientific challenges that are still open and provide interesting opportunities for further research and progress.

